# Genome-wide association mapping of iron homeostasis in the maize association population

**DOI:** 10.1186/s12863-014-0153-0

**Published:** 2015-01-30

**Authors:** Andreas Benke, Claude Urbany, Benjamin Stich

**Affiliations:** Max Planck Institute for Plant Breeding Research, Carl-von-Linné Weg 10, 50829 Köln, Germany

**Keywords:** Fe-efficiency, Association mapping population, Fine-mapping, Genome-wide association, Marker assisted selection

## Abstract

**Background:**

Iron (Fe) deficiency in plants is the result of low Fe soil availability affecting 30% of cultivated soils worldwide. To improve our understanding on Fe-efficiency this study aimed to (i) evaluate the influence of two different Fe regimes on morphological and physiological trait formation, (ii) identify polymorphisms statistically associated with morphological and physiological traits, and (iii) dissect the correlation between morphological and physiological traits using an association mapping population.

**Results:**

The fine-mapping analyses on quantitative trait loci (QTL) confidence intervals of the intermated B73 × Mo17 (IBM) population provided a total of 13 and 2 single nucleotide polymorphisms (SNPs) under limited and adequate Fe regimes, respectively, which were significantly (FDR = 0.05) associated with cytochrome P450 94A1, invertase beta-fructofuranosidase insoluble isoenzyme 6, and a low-temperature-induced 65 kDa protein. The genome-wide association (GWA) analyses under limited and adequate Fe regimes provided in total 18 and 17 significant SNPs, respectively.

**Conclusions:**

Significantly associated SNPs on a genome-wide level under both Fe regimes for the traits leaf necrosis (NEC), root weight (RW), shoot dry weight (SDW), water (H _2_O), and SPAD value of leaf 3 (SP3) were located in genes or recognition sites of transcriptional regulators, which indicates a direct impact on the phenotype. SNPs which were significantly associated on a genome-wide level under both Fe regimes with the traits NEC, RW, SDW, H _2_O, and SP3 might be attractive targets for marker assisted selection as well as interesting objects for future functional analyses.

**Electronic supplementary material:**

The online version of this article (doi:10.1186/s12863-014-0153-0) contains supplementary material, which is available to authorized users.

## Background

Iron (Fe) deficiency in plants is the result of a low Fe availability which might be induced by lime-chlorosis that affects 30% of cultivated soils worldwide [1]. As an adaptation to the sparingly available Fe, plants evolved two different strategies to mobilize and uptake Fe [2]. Dicotyledonous and non graminaceous plant species acquire Fe by the so-called strategy I mechanism [3]. The characteristic of this strategy is the release of protons into the rhizosphere that facilitate the mobilization and subsequent reduction of Fe(III) to Fe(II) via a plasma membrane bound Fe(III) chelate reductase [4]. The soluble Fe(II) is finally taken up by the iron regulated transporter 1 (IRT1) [5].

For the crop plants which are graminaceous plant species such as barley, rice, and maize, Fe is acquired using the so-called strategy II [6]. Characteristic for this strategy is the release of non proteinogenic compounds named phytosiderophores. These compounds chelate the Fe(III) in the rhizosphere. Phyto-siderophore-Fe(III) complexes are transported by the specific transporter yellow stripe 1 (YS1) into the plant [7]. It was shown by [2] that the amount of exudated phytosiderophores is crucial for a chlorosis tolerance and therefore, Fe-efficient plant. However, for an Fe-efficient genotype, the balance of Fe dependent systems like Fe mobilization and uptake into the plant and the homeostasis related mechanisms like translocation and regulation of the Fe level in the cell to avoid shortage or toxicity [8,9] is essential.

To improve our understanding of the mechanisms which are responsible for Fe-efficiency in maize, two different methods have been applied so far. The RNA-Sequencing approach used by [10] focused on genes which were differentially expressed between the Fe-efficient and inefficient inbred lines under sufficient and deficient Fe regimes. This study provided a tremendous amount of putative candidate genes for Fe-efficiency. The same inbred lines were used for the establishment of the intermated B73 × Mo17 (IBM) segregating population [11]. Benke et al., 2014 [12] observed a considerable phenotypic variation for Fe-efficiency in this population which was used to map quantitative trait loci (QTL). An alternative to linkage mapping is association mapping which has the potential to provide a higher mapping resolution as well as allows the evaluation of a higher number of alleles at a time. To our knowledge, no genome-wide association study has been conducted to dissect Fe-efficiency in maize.

The objectives of our study were to (i) evaluate the influence of different Fe regimes on morphological and physiological trait formation, (ii) identify polymorphisms statistically associated with morphological and physiological traits, and (iii) dissect the correlation between morphological and physiological traits using an association mapping population.

## Results

The repeatability (*H*^2^) of the examined traits ranged for the whole set of phenotyped inbred lines from 0.53 (H _2_O) to 0.72 (SP3, SP4, and RL) under the Fe-deficient regime (Table 1). *H*^2^ of the traits evaluated under the Fe-sufficient regime varied between 0.47 (H _2_O) and 0.87 (SP4).

**Table 1:** **Traits recorded in the current study for two deficient and sufficient iron (Fe) regimes, where** ***H*** ^***2***^ **is the repeatability on an entry means basis for the association mapping population**

			***H*** ^**2**^	
**Trait**	**Abbreviation**	**Unit**	**Fe-deficient**	**Fe-sufficient**
SPAD value at leaf 3	SP3	SPAD units	0.72	0.86
SPAD value at leaf 4	SP4	SPAD units	0.72	0.87
SPAD value at leaf 5	SP5	SPAD units	0.68	0.81
SPAD value at leaf 6	SP6	SPAD units	0.68	0.77
Root length	RL	cm	0.72	0.62
Root weight	RW	g	0.59	0.47
Shoot length	SL	cm	0.63	0.57
Shoot dry weight	SDW	g	0.65	0.65
Shoot water content	H _2_O	%	0.53	0.44
Ratio of dry shoot weight	SDW/SL	g/cm	0.68	0.71
compared to shoot length				
Branching at the terminal 5 cm	BTR	score 1 - 9	0.68	^1^
Lateral root formation	LAT	score 1 - 9	0.68	0.58
Leaf necrosis	NEC	score 1 - 9	0.61	0.71

^1^no variation observed.

**Figure 1:**
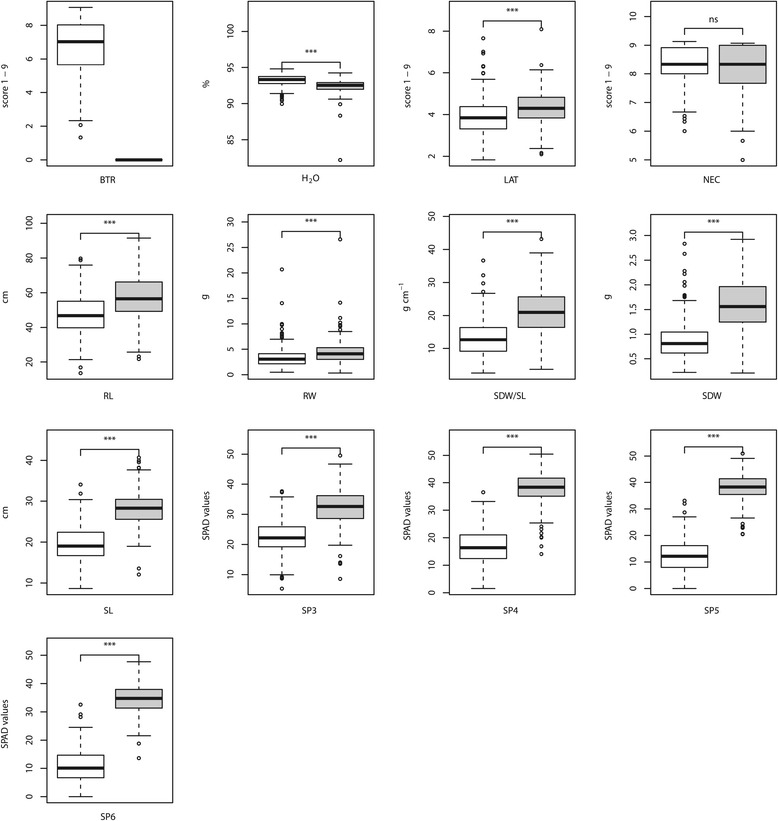
**Boxplot of the adjusted entry means for the association mapping population of 267 maize inbred lines evaluated at Fe-deficient and Fe-sufficient regimes represented in white and gray, respectively.** T-test was applied to examine the difference of a trait between both Fe conditions. ***: *P* = 0.05, 0.01, and 0.001, respectively; ns, not significant.

The adjusted entry means (AEM) were calculated for all physiological and morphological traits under consideration of the block effects for each Fe regime (Figure 1). No variation was observed for BTR under the Fe-sufficient regime. For NEC, no significant (*α*= 0.05) difference between both Fe regimes was found. The remaining morphological and physiological traits except H _2_O showed a significant (*α*= 0.05) lower trait value under the Fe-deficient regime in comparison to the Fe-sufficient regime. For H _2_O the opposite trend was observed.

**Figure 2:**
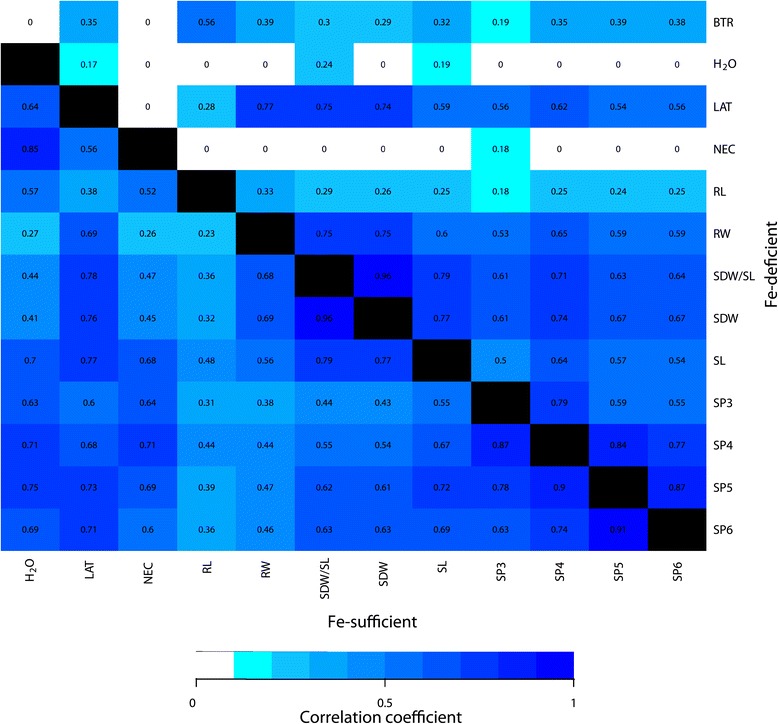
**Pairwise correlation coefficients calculated between all pairs of traits collected for the association mapping population.** The values above the diagonal represent the correlation coefficients between the adjusted entry means (AEM) of the Fe-deficient regime. The values below the diagonal represent the correlation coefficients between the AEM of the Fe-sufficient regime.

The lowest pairwise correlation coefficient was with *r* = 0.17 observed between H _2_O and LAT under the Fe-deficient regime (Figure 2). By comparison, for the Fe-sufficient regime, the higher positive correlation coefficient was observed between SDW/SL and SDW (*r* = 0.96) and the lowest between RL and RW (*r* = 0.23).

In the ASMP, the population structure explained on average 2.02% of the phenotypic variation with a minimum of 0.08% (SL) and a maximum of 5.32% (RL) under the Fe-deficient regime (Additional file 1: Table S1). Under the Fe-sufficient regime, the population structure accounted on average for 2.42% of the phenotypic variation ranging from 0.35% (SDW) to 5.09% (RL).

**Figure 3:**
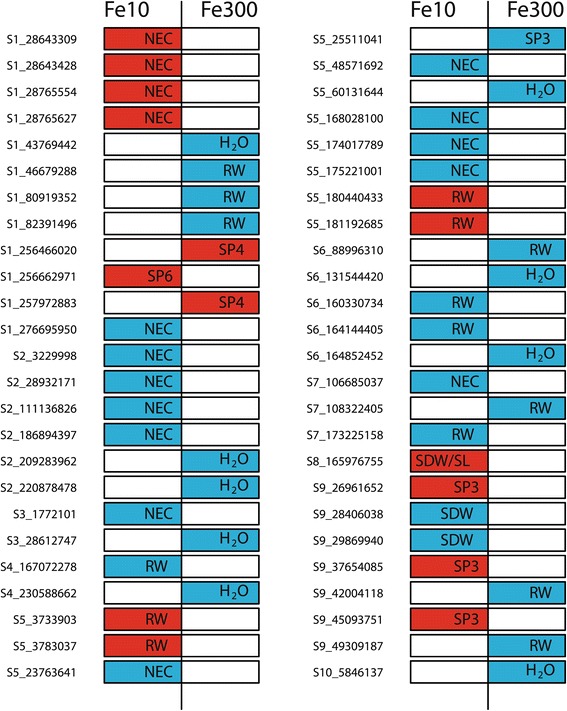
**Summary of significant (FDR = 0.05) single nucleotide polymorphisms (SNPs) detected in confidence intervals of quantitative trait loci (QTL) (red) of [** 12 **] and genome-wide SNPs association analyses (blue) using the association mapping population with respect to the iron (Fe) regime 10** ***μ*** **M and 300** ***μ*** **M.**

The QTL fine-mapping (FM) analyses resulted in total in 13 significant (FDR = 0.05) SNPs detected in QTL confidence intervals of the IBM population where NEC QTL1 comprised the highest amount (4) under the Fe-deficient regime (Table 2, Figure 3). The highest proportion of phenotypic variance was explained by a SNP in QTL3 of RW (8.47%). The maximum proportion of phenotypic variance explained in a simultaneous fit by all SNPs in a QTL confidence interval was 11.45% (QTL8 SP3) and the minimum was 0.39% (QTL4 RW).

**Table 2:** **Single nucleotide polymorphism (SNP) markers significantly (FDR = 0.05) associated in the association mapping population which were located within confidence intervals of QTL detected for the same trait in the IBM population [** 12 **]**

			**Marker**		**Position**	**Interval**		**Allele**	**Effect**			**Best hit**	**Confirmed**
**Fe regime**	**Trait**	**QTL**	**locus**	**Chr.**	**(bp)**	**(cM)**	***P*** **-value**	**1/2**	**Allele 1-2**	**% r** ^**2**^	**Gene**	**UniProt ID**	**Annotation**
Deficient	NEC	QTL1	S1_28643309	1	28,643,309	205.0 - 208.5	8.2e-05	G/A	0.43	6.04	GRMZM2G040828	Q9LVS3	Pentatricopeptide repeat-containing protein At5g47360
			S1_28643428	1	28,643,428	205.0 - 208.5	1.7e-04	G/A	0.41	5.43	GRMZM2G040828	Q9LVS3	Pentatricopeptide repeat-containing protein At5g47360
			S1_28765554	1	28,765,554	205.0 - 208.5	1.7e-04	A/G	0.42	5.51	GRMZM2G036257	O81117	Cytochrome P450 94A1
			S1_28765627	1	28,765,627	205.0 - 208.5	1.5e-04	G/A	0.39	4.96	GRMZM2G036257	O81117	Cytochrome P450 94A1
			Simultaneous fit							5.29			
	RW	QTL3	S5_3733903	5	3,733,903	73.3 - 74.4	2.1e-04	C/T	-3.87	8.47	GRMZM2G701295		
											GRMZM2G350471	Q75HK3	Expressed protein
			S5_3783037	5	3,783,037	73.3 - 74.4	1.2e-03	C/G	0.60	0.18	GRMZM2G350428	Q6AV48	CLE family OsCLE305 protein
			Simultaneous fit							8.67			
	RW	QTL4	S5_180440433	5	180,440,433	410.8 - 413.6	3.0e-04	C/G	0.27	0.04			
			S5_181192685	5	181,192,685	410.8 - 413.6	2.3e-04	T/C	0.24	0.03	AC205703.4_FG005	Q8GUM4	Uncharacterized membrane protein At3g27390
			Simultaneous fit							0.39			
	SDW/SL	QTL1	S8_165976755	8	165,976,755	464.0 - 466.5	6.0e-05	C/T	-7.09	6.87	GRMZM2G148773		
			Simultaneous fit							6.87			
	SP3	QTL8	S9_26961652	9	26,961,652	220.7 - 223.9	5.7e-06	A/T	5.85	7.92	GRMZM2G156218	F4K975	Sec14p-like phosphatidylinositol transfer family protein
			S9_37654085	9	37,654,085	220.7 - 223.9	1.4e-04	C/T	6.16	5.56			
			S9_45093751	9	45,093,751	220.7 - 223.9	4.6e-05	A/G	4.20	6.78	GRMZM2G177084	Q9FG31	Late embryogenesis abundant protein 4-5
			Simultaneous fit							11.45			
	SP6	QTL3	S1_256662971	1	256,662,971	825.8 - 833.0	1.2e-04	G/A	9.88	5.82			
			Simultaneous fit							5.82			
Sufficient	SP4	QTL1	S1_256466020	1	256,466,020	833.0 - 839.3	7.8e-05	T/C	3.66	6.32			
			S1_257972883	1	257,972,883	833.0 - 839.3	1.1e-04	T/A	5.45	6.05	GRMZM2G055037	Q3E8H0	S-ribonuclease binding protein
			Simultaneous fit							10.31			

% r ^2^ is the proportion of the phenotypic variance explained by the SNP for the association mapping population.

Under the Fe-sufficient regime, the QTL FM analyses revealed in total two significant (FDR = 0.05) SNPs for SP4 QTL1 (Table 2, Figure 3). The maximum proportion of phenotypic variance of SNPs was 6.32%. The phenotypic proportion was 10.31% for both SNPs in a simultaneous fit.

The genome-wide association (GWA) analyses of the traits examined in the Fe-deficient regime provided in total 18 significant SNPs (FDR = 0.05) where NEC showed with 12 SNPs the highest number (Table 3, Figure 3, Additional file 2: Figure S1;A, Additional file 3: Figure S3;A). The proportion of phenotypic variance explained by a SNP showed for RL (18.81%) the highest value. The proportion of phenotypic variance explained in a simultaneous fit by all SNPs for one trait was maximal for RW (34.65%) and minimal for SDW (13.01%).

**Table 3:** **Single nucleotide polymorphism (SNP) markers significantly (FDR = 0.05) associated with traits evaluated under Fe-deficient and the Fe-sufficient iron regime**

		**Marker**		**Position**		**Allele**	**Effect**			**Best hit**	**Confirmed**
**Fe regime**	**Trait**	**locus**	**Chr.**	**(bp)**	***P*** **-value**	**1/2**	**Allele 1-2**	**% r** ^**2**^	**Gene**	**UniProt ID**	**Annotation**
Deficient	NEC	S1_276695950	1	276,695,950	7.0e-07	G/A	0.91	10.58			
		S2_3229998	2	3,229,998	9.4e-08	T/A	1.51	12.39	GRMZM2G018692	Q56UD0	Beta-fructofuranosidase, insoluble isoenzyme 6
		S2_28932171	2	28,932,171	4.4e-08	A/T	1.55	13.03			
		S2_111136826	2	111,136,826	1.5e-06	A/G	1.16	10.08			
		S2_186894397	2	186,894,397	9.0e-07	C/T	0.81	10.63	GRMZM2G168163	Q9SX79	Polyadenylate-binding protein RBP47C
		S3_1772101	3	1,772,101	7.4e-07	A/G	0.83	10.89			
		S5_23763641	5	23763641	1.4e-06	C/G	1.04	10.50	GRMZM2G376743	Q04980	Low-temperature-induced 65 kDa protein
		S5_48571692	5	48,571,692	1.5e-09	T/C	1.16	14.68	GRMZM5G848124	Q851X4	Expressed protein
		S5_168028100	5	168,028,100	1.1e-06	C/G	1.34	10.08			
		S5_174017789	5	174,017,789	1.6e-07	C/T	0.89	11.09	GRMZM2G460958	Q9LRB7	E3 ubiquitin-protein ligase EL5
		S5_175221001	5	175,221,001	1.2e-07	T/G	1.28	11.52	GRMZM2G128029	Q2R2T4	CASP-like protein Os11g0549625
		S7_106685037	7	106,685,037	1.0e-06	T/G	1.21	10.05			
		Simultaneous fit						30.78			
	RW	S4_167072278	4	167,072,278	3.7e-10	C/A	-6.23	18.81	GRMZM2G015049	Q9LR00	SAUR-like auxin-responsive protein
		S6_160330734	6	160,330,734	1.5e-07	C/G	-3.67	13.63			
		S6_164144405	6	164,144,405	6.0e-07	T/C	-4.78	12.73	GRMZM2G377613	P23923	Transcription factor HBP-1b(c38)
		S7_173225158	7	173,225,158	4.4e-07	G/T	-4.31	13.69	GRMZM2G381386	F4II36	RING-finger, DEAD-like helicase, PHD and SNF2 domain
		Simultaneous fit						34.65			
	SDW	S9_28406038	9	28,406,038	7.0e-10	C/T	-0.72	16.35			
		S9_29869940	9	29,869,940	1.7e-07	T/C	-0.62	11.44			
		Simultaneous fit						13.01			
Sufficient	H _2_O	S1_43769442	1	43,769,442	1.6e-06	A/G	3.00	13.52			
		S10_5846137	10	5,846,137	5.4e-07	C/A	3.59	14.81			
		S2_209283962	2	209,283,962	1.4e-06	G/T	2.27	14.06			
		S2_220878478	2	220,878,478	4.7e-08	C/T	2.99	16.30			
		S3_28612747	3	28,612,747	7.7e-07	T/C	2.75	14.90			
		S4_230588662	4	230,588,662	1.2e-06	G/A	1.91	14.11	GRMZM2G038588	Q54N48	Protein CLP1 homolog
		S5_60131644	5	60,131,644	1.3e-06	A/T	2.38	13.80	GRMZM2G097683	Q9XGX0	Putative zinc finger protein SHI
		S6_131544420	6	131,544,420	8.4e-12	T/C	5.04	21.21			
		S6_164852452	6	164,852,452	7.2e-07	G/A	-0.10	0.16	GRMZM2G030305	Q5SN53	Mitogen-activated protein kinase 8
		Simultaneous fit						57.47			
	RW	S1_46679288	1	46,679,288	3.3e-09	C/G	-12.59	20.69	GRMZM2G455809	P50160	Sex determination protein tasselseed-2
		S1_80919352	1	80,919,352	6.4e-08	A/C	-9.18	17.60	GRMZM2G087878		
		S1_82391496	1	82,391,496	2.5e-10	G/A	-12.76	20.72			
		S6_88996310	6	88,996,310	6.7e-07	C/T	-7.87	15.38	GRMZM2G439598		
		S7_108322405	7	108,322,405	1.8e-06	T/C	0.58	0.15			
		S9_42004118	9	42,004,118	9.8e-07	G/A	0.63	0.86			
		S9_49309187	9	49,309,187	5.4e-07	C/G	-9.48	15.71	GRMZM2G040605		
		Simultaneous fit						38.93			
	SP3	S5_25511041	5	25,511,041	7.9e-08	A/C	-4.41	10.99	GRMZM2G305446	Q9ATL7	Aquaporin TIP3-1
		Simultaneous fit						10.99			

% r ^2^ is the proportion of the phenotypic variance explained by the SNP for the association mapping population.

The GWA analyses under the Fe-sufficient regime revealed in total 17 significant (FDR = 0.05) SNPs where H _2_O (9) included the highest number (Table 3, Figure 3, Additional file 4: Figure S2;A, Additional file 5: Figure S4; A). The proportion of the explained phenotypic variance was highest for H _2_O (21.21%). In a simultaneous fit of all significant (FDR = 0.05) SNPs, proportion of the phenotypic variance maximally explained was 57.47% (H _2_O) and the minimum was 10.99% (SP3).

Under consideration of the global extent of LD, 18 and 9 unique genes were linked to the significantly (FDR = 0.05) associated SNPs under the Fe-deficient and Fe-sufficient regime, respectively (Tables 2 and 3). None of the Sanger-sequenced genes evaluated in Additional file 2: Figure S1 included SNPs that were significantly (FDR = 0.05) associated with the morphological and physiological traits.

## Discussion

Environmental factors such as pH variation in the soil, temperature, water stress, and mineral concentration effects have a strong influence on Fe availability for plants [2]. To reveal genotypic effects that contribute to Fe-efficiency and avoid an overlap with other mineral nutrients, hydroponic culture has been proven to be the method of choice providing standard environmental conditions [13]. Such a culture has been used in our study to examine the Fe-efficiency in a broad germplasm set of maize.

### Dissection of phenotypic diversity and relation between the examined traits

We observed for all traits moderate to high repeatabilities under both Fe regimes (Table 1). This finding indicated that the genetic contribution to variation was minimally covered by experimental variation of hydroponics which in turn increases the power of the genetic dissection of Fe-efficiency by association mapping methods.

We observed, under the Fe-deficient regime, variation for the trait BTR (Figure 1). Long et al. 2010 [14] revealed an Fe sensing gene named POPEYE in Arabidopsis roots during Fe-deficiency. Their finding indicated that Fe deficiency sensing mechanisms regulate terminal root branching. However, in contrast to Arabidopsis [14], in maize the mechanism of root branching under Fe-deficiency is not yet understood.

The whole set of traits evaluated in one Fe regime showed mostly moderate to high pairwise correlations (Figure 2). This finding suggests that for each of the Fe-sufficient and Fe-deficient regimes most of the examined traits have a joint regulation. One of the few exception was the correlation between leaf necrosis and water content, which was only observed in the Fe-sufficient regime. This positive correlation might be caused by a nutrient distortion, also known as concentration effect [2].

### Marker-phenotype associations for QTL confidence intervals and on genome-wide scale

Using the ASMP we were able to validate 13% and 3% of detected QTLs from our former study [12] for Fe-deficient and Fe-sufficient regimes, respectively. Among the SNPs that were located within QTL confindence intervals [12], we identified a SNP (S1_28765627) in the cytochrome P450 94A1 (CYP94A1) (GRMZM2G036257) gene that was significantly associated with NEC (Table 2). CYP94A1 is responsible for modifying lipophilic compounds like fatty acids [15]. Its involvement in plant development, repair, and defense [15] might indicate the contribution of stress response mechanisms during Fe-deficiency. Furthermore, cytochrome P450 family proteins might also play a role in Fe sensing [16] as Fe is incorporated into a heme group of the cytochrome P450 proteins [17].

**Figure 4:**
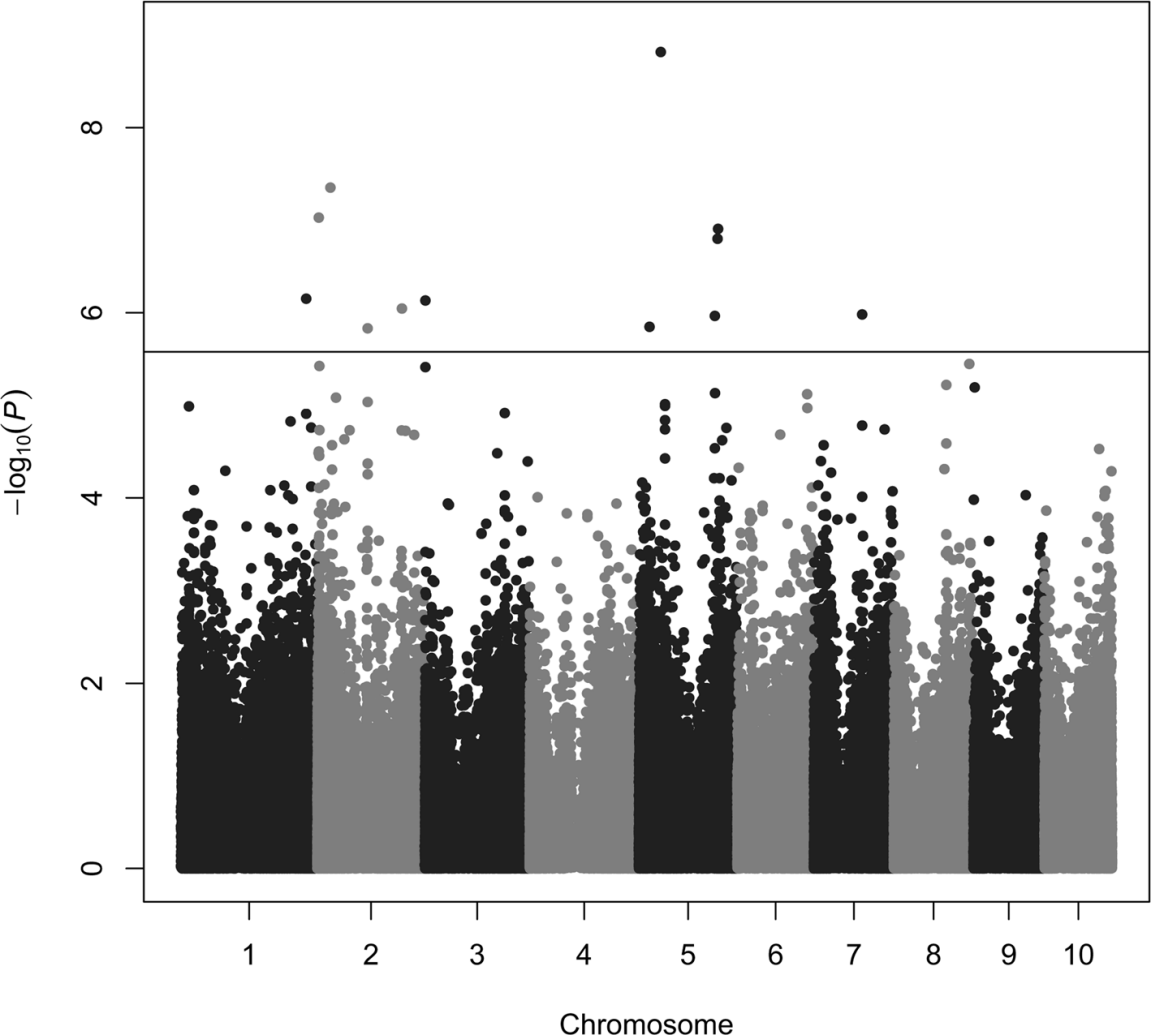
**Genome-wide** ***P*** **values for association analysis of NEC under the Fe-deficient regime using 267 maize inbred lines of the association mapping population.** The horizontal line corresponds to a nominal significance threshold of 5% considering the Benjamini Hochberg correction for multiple testing.

We observed under the Fe-deficient regime several genes to be associated with NEC (Figure 4) and RW that are mechanistically involved in regulation of stress response (Table 3). A subset of these genes includes the invertase beta-fructofuranosidase insoluble isoenzyme 6 (NEC,GRMZM2G018692) [18], low-temperature-induced 65 kDa protein (NEC,GRMZM2G376743) [19], and the late embryogenesis abundant protein 4-5 (SP3,GRMZM2-G177084) [20]. O’Rourke et al., 2007 [21] showed that these genes are responsible for the universal stress response caused by Fe-deficiency, although they do not bind or incorporate Fe in their protein structure. This suggested that these genes are important to maintain the viability of the plant due to stress prevention caused by Fe-deficiency. Furthermore, significant associations for NEC might indicate that this trait is genetically less complex than Fe-chlorosis as for the SPAD value related traits no significant association could have been detected under the Fe-deficient regime.

We did not observe a clear clustering of genotypes with high NEC values in the individual subgroups. Furthermore, when examing the subgroups individually (Additional file 1: Table S1), we detected no significant associations neither for NEC nor for RW under both Fe regimes (data not shown). Additionally, excluding genotypes with a higher NEC susceptibility from the association analysis changed the results only marginally compared to the analyses with all genotypes. These results suggested that the concentration effect does not influence the conclusions of our study.

Despite the variation observed for BTR under the Fe-deficient regime, no significant associations have been detected. Therefore, further research is required on the genetics of BTR. In that context, the genes identified in our companion study [10] using an RNA sequencing approach can be promising starting points.

In our study, genes, known being mechanistically involved in strategy II related processes for Fe mobilization, uptake and storage, were resequenced (Additional file 6: Table S2). For polymorphisms in these genes, no significant associations were detected for both Fe regimes. This finding could be explained by a correlation of allele frequency of the mechanistically involved genes and population structure as was observed previously for flowering time and *Dwarf8* [22,23]. As we did not observe a strong correlation between population structure and phenotypic variation of the studied traits this explanation is not likely to be true (Additional file 1: Table S1). The reason could be that these mechanistically involved genes have been identified by mutant screening only and that natural genetic variation at these genes leads to evolutionary disadvantages. Therefore, only neutral polymorphisms with respect to the phenotype are observed in the maize ASMP. This might reflect purified selection of these adaptive genes that does not contribute to phenotypic variation of quantitative trait [24].

An overlap between associated SNPs of traits were not observed putatively due to minor effect associations and a stringent significance thresholds applied in our study. Nevertheless, significant association of SNPs and their corresponding genes as described above provide an insight in the genetic architecture of biological processes characteristic for each trait that is in a direct relation to Fe-homeostasis. However, association mapping analyses provide only an indirect statistical evidence for a contribution of the considered allele to phenotypic variation [25] a direct functional validation is indispensable. Furthermore, additional traits like protein and transcriptome expression profiling could be performed on the association mapping population to further dissect Fe-homeostasis.

## Conclusions

The QTL confidence intervals of the traits NEC, RW, SDW/SL, SP3, SP4, and SP6, from a previous study contained hundreds of genes and millions of base pairs. A dissection of these QTL confidence intervals using association mapping methods allowed a confirmation of the previously detected QTLs as well as the fine-mapping. In addition, our study described SNPs which were significantly associated on a genome-wide level under both Fe regimes with the traits NEC, RW, SDW, H _2_O, and SP3. Several of these SNPs were located in genes (coding) or recognition sites (non-coding) of transcriptional regulators, which indicates a direct impact on the phenotype. Beside being attractive targets for marker assisted selection, these loci are interesting objects for future functional analyses.

## Methods

### Plant material

A set of 302 maize inbred lines representing world-wide maize diversity [26] was used as association mapping population (ASMP) in the current study. Due to the unavailability of sufficient amounts of seeds for 35 inbred lines, a final set of 267 inbred lines was evaluated in the frame of this study (Additional file 7: Table S4).

### Culture conditions and evaluated traits

Maize seeds were sterilized with 60°C hot water for 30 minutes. Afterwards, seeds were placed between two filter paper sheets moistened with saturated CaSO _4_ solution for germination in the dark at room temperature. After 6 days, the germinated seeds were transplanted to a continuously aerated nutrient solution with nutrient concentrations as described by [27]. The plants were supplied with 100 *μ*M Fe(III)-EDTA for 7 days. From day 14 to 28, plants were cultured at 10 (Fe-deficient) and 300 (Fe-sufficient) *μ*M iron regimes. The nutrient solution was exchanged every third day. Plants were cultivated from day 7 to day 28 in a growth chamber at a relative humidity of 60%, light intensity of 170 *μ*mol m ^−2^ s ^−1^ in the leaf canopy, and a day-night temperature regime of 16 h/24°C and 8 h/22°C, respectively.

Each genotype was grown in one shaded pot of 600 milliliter volume. All pots of one Fe regime were arranged in an alpha lattice design with 13 incomplete blocks. The entire experiment was replicated *b*= 3 times for the Fe-deficient and sufficient regime, respectively.

Under both Fe regimes, the following traits were evaluated: the relative chlorophyll content of the 3rd, 4th, 5th, and 6th leaf (SP) measured with a SPAD meter (Minolta SPAD 502). Branching at the terminal 5 cm of the root (BTR) was evaluated with 1 for strong presence and 9 for absence of terminal root branching. Leaf necrosis (NEC) was recorded as a visual score on a scale from 1 for high trait expression and 9 for low trait expression. The lateral root formation (LAT) was recorded on a scale from 1 for absence to 9 for high trait expression. Furthermore, root length (RL), root weight (RW), shoot length (SL), shoot dry weight (SDW), water content (H _2_O) as well as the ratio between SDW and SL (SDW/SL) was according to [12].

In our study, the data collected in this way for both Fe regimes were not directly combined to calculate a response variable for each trait in order to avoid problems related to error propagation. Instead, we followed examples from the literature and analysed data from the regimes individually but compared the results afterwards.

### SNP marker data

A data set with 437,650 SNP markers for the ASMP is publicly available from http://www.panzea.org. If for one SNP more than 20% of the marker information across all inbreds was unknown or denoted as missing data, this mSNP was skipped from the following analyses. Furthermore, SNPs with minor allele frequency lower than 2.5% were excluded from the following analyses.

### Sequence analysis

A set of 16 candidate genes for mobilization, uptake, storage, and transport of Fe as well as regulatory function on these processes was selected for sequence analyses to detect additional polymorphisms compared to the above mentioned SNP data set (Additional file 2: Figure S1). Primers for candidate genes were designed using software Primer3 [28] (Additional file 8: Table S3). Each region of the candidate gene sequence was PCR amplified for the ASMP. PCR products were sequenced by the DNA core facility of the Max-Planck-Institute for Plant Breeding Research on Applied Biosystems (Weiterstadt, Germany) Abi 3730XL sequencers using BigDye-terminator v3.1 chemistry. Premixed reagents were from Applied Biosystems. The gene sequences were aligned with the software ClustalW2 (http://download.famouswhy.com/clustalw2/) and edited with BioLign (http://en.bio-soft.net/dna/BioLign.html) manually. The SNPs were filtered as described above and the remaining 562 SNPs were added to the above mentioned set of genome-wide distributed SNPs.

### Statistical analyses

*Phenotypic data analyses:* The traits collected at each Fe regime were analyzed using the following mixed model:

$$y_{ikm}= \mu + g_{i} + r_{k}+ b_{km} + e_{ikm}{,} $$

where *y*_*ikm*_ is the *i*th genotype of the *k*th replication in the *m*th incomplete block, *μ* the general mean, *g*_*i*_ the effect of the *i*th genotype, *r*_*k*_ the effect of the *k*th replication, *b*_*km*_ the effect of the *m*th incomplete block in the *k*th replication, and *e*_*ikm*_ the residual error. To estimate adjusted entry means (AEM) for all inbreds at each of two Fe regimes, we considered *g* as fixed as well as *r* and *b* as random. Furthermore, we considered *g*, *r*, and *b* as random to estimate the genotypic (${\sigma }^{2}_{g}$) and the error variance (${\sigma }^{2}_{e}$).

The repeatability *H*^2^ for each Fe regime was calculated as:

$$H^{2}= \frac{{\sigma^{2}_{g}}}{{\sigma^{2}_{g}} + \frac{{\sigma^{2}_{e}}}{b}}{.} $$

The residuals for each trait under both Fe regimes were tested with a Kolmogorov-Smirnov test [29] for their normal distribution. Pairwise correlation coefficients were assessed between all pairs of traits for the ASMP. Student’s t-tests were calculated for each trait to examine the significance of the difference between the Fe-deficient and sufficient regimes.

*Association analyses:* The AEM of each trait for each Fe regime were used to test their associations with each of the 287,390 SNP markers using the following mixed model:

$$M_{ip}= \mu + m_{p} + g^{*}_{i} + \sum^{z}_{u=1} Q_{iu}v_{u} + e_{ip}{,} $$

where *M*_*ip*_ is the AEM of the *i*th maize inbred line carrying the *p*th allele, *m*_*p*_ the effect of allele *p*, *g*^∗^_*i*_ the residual genetic effect of the *i*th inbred line, *v*_*u*_ the effect of the *u*th column of the population structure matrix Q [26], and *e*_*ip*_ the residual [30]. The variance-covariance matrix of the vector of random effects *g*^∗^=*g*^∗^_1_,…,*g*^∗^_267_ was assumed to be *Var*(*g*^∗^) = 2K$\sigma ^{2}_{g^{*}}$, where K was a 267 × 267 matrix of kinship coefficients among the ASMP [31], and $\sigma ^{2}_{g^{*}}$ genetic variance estimated by REML. The relation between the population structure and the morphological and physiological traits was estimated using the ‘EMMA’ R package [31].

Physical map positions of QTL confidence intervals detected in the linkage mapping study of [12] were used for fine-mapping.

Multiple testing was considered by applying the [32] correction. The proportion of phenotypic variation explained by the significant SNPs was computed according to [33].

For each SNP of the marker set, the information about the physical map position was available. The extent of linkage disequilibrium in the maize ASMP which was estimated by [34] was used to determine the genes which are linked to the detected SNP in the association analysis: up and downstream of a significant association the genes included in the region 2,000 base pairs were extracted from the filtered gene set of the maize genome sequence version 5b.

If not stated differently, all analyses were performed using statistical software R [35].

## Additional files

Additional file 1
**Table S1.** Phenotypic variation (%r ^2^) explained by population structure and by kinship for the entire association mapping population set.

Additional file 2
**Figure S1.** Genome-wide *P* values for association analysis under the Fe-sufficient regime using 267 maize inbred lines of the association mapping population. The horizontal line corresponds to a nominal significance threshold of 5% considering the Benjamini Hochberg correction for multiple testing. Traits with significant SNPs are represented: shoot water content (H _2_O;A), root weight (RW;B), and SPAD value of leaf 3 (SP3;C).

Additional file 3
**Figure S3.** Expected *P* values on the horizontal axis and observed *P* values on the vertical axis for the QQ plot analysis under the Fe-sufficient regime using 267 maize inbred lines of the association mapping population. The red line corresponds to a normal distribution. Traits with significant SNPs are represented: shoot water content (H _2_O;A), root weight (RW;B), and SPAD value of leaf 3 (SP3;C).

Additional file 4
**Figure S2.** Expected *P* values on the horizontal axis and observed *P* values on the vertical axis for the QQ plot analysis under the Fe-deficient regime using 267 maize inbred lines of the association mapping population. The red line corresponds to a normal distribution. Traits with significant SNPs are represented: leaf necrosis (NEC;A), root weight (RW;B), and shoot dry weight (SDW;C).

Additional file 5
**Figure S4.** Genes sequenced in our study that are reported in the literature to be involved in Fe-homeostasis of maize.

Additional file 6
**Table S2.** List of 267 maize genotypes comprising the source history, pedigree information, and assigned subpopulation.

Additional file 7
**Table S4.** Primer list (forward: F; reverse: R) of sequenced amplicons with base pair (bp) length in B73. The annealing temperature (An. Temp) was empirically determined.

Additional file 8
**Table S3.** Genome-wide *P* values for association analysis under the Fe-deficient regime using 267 maize inbred lines of the association mapping population. The horizontal line corresponds to a nominal significance threshold of 5% considering the Benjamini Hochberg correction for multiple testing. Traits with significant SNPs are represented: leaf necrosis (NEC;A), root weight (RW;B), and shoot dry weight (SDW;C).
