# Supplementary figures and images for: Genome-wide association mapping of iron homeostasis in the maize association population
Source: BMC Genet. 2015 Jan 30;16:1. doi: 10.1186/s12863-014-0153-0 (PMC4342226; doi:10.1186/s12863-014-0153-0)

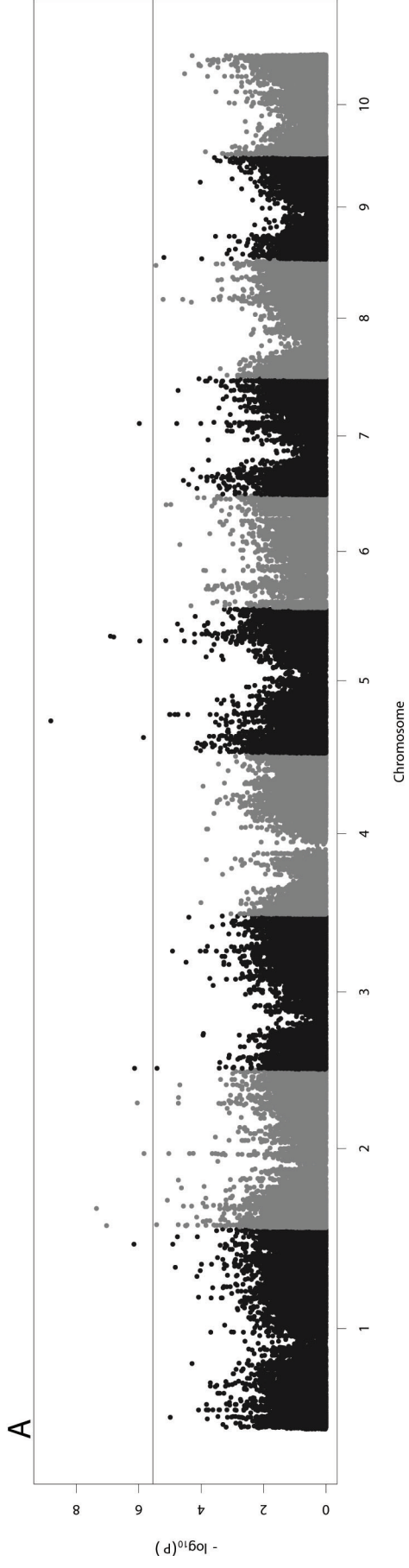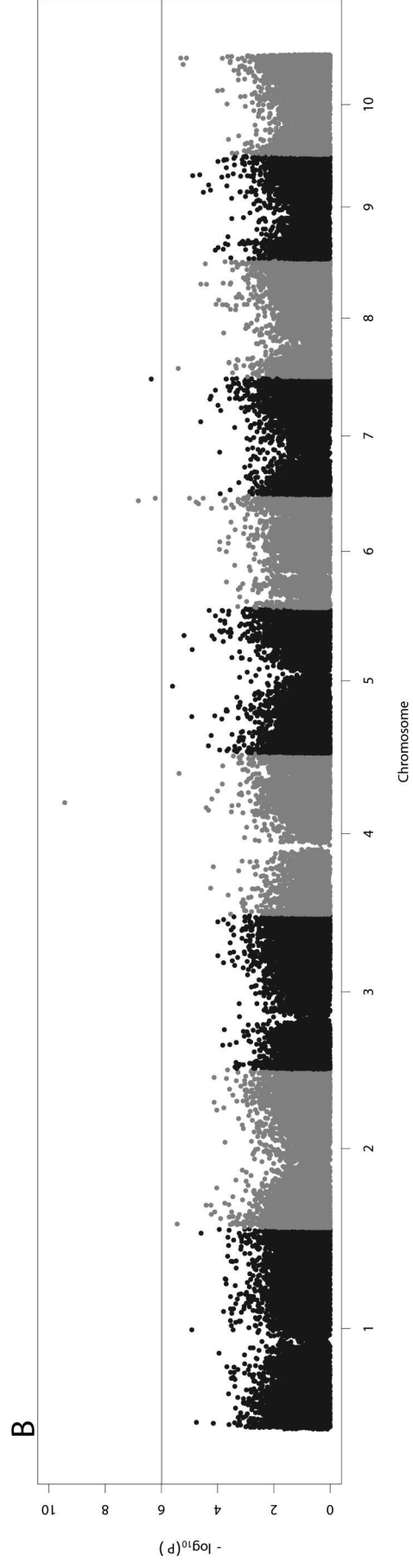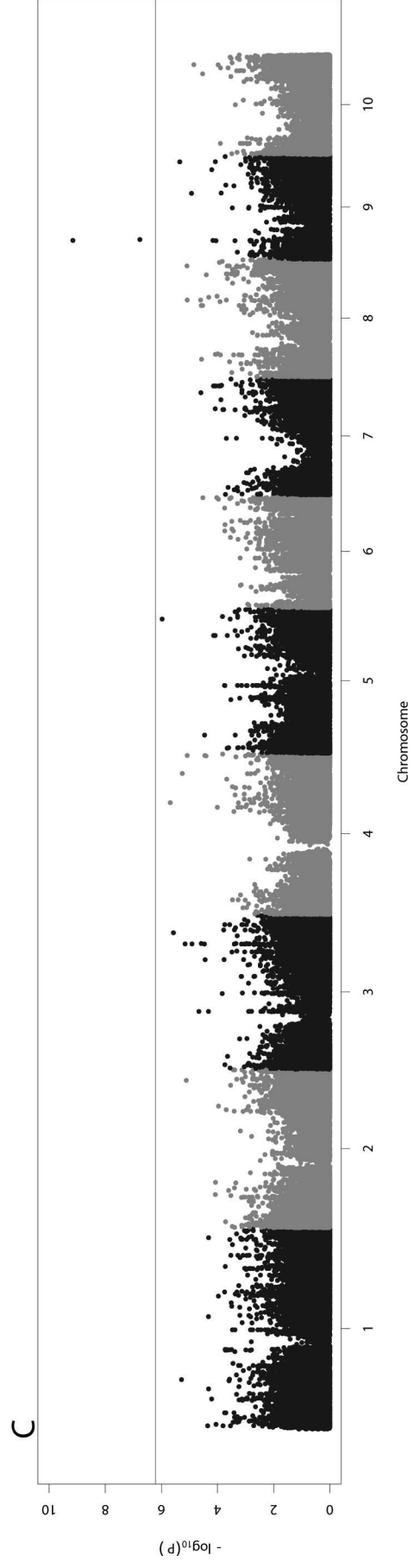

Supplement: Additional file 2 — Figure S1. Genome-wide P values for association analysis under the Fe-sufficient regime using 267 maize inbred lines of the association mapping population. The horizontal line corresponds to a nominal significance threshold of 5% considering the Benjamini Hochberg correction for multiple testing. Traits with significant SNPs are represented: shoot water content (H 2O;A), root weight (RW;B), and SPAD value of leaf 3 (SP3;C). [file 12863_2014_153_MOESM2_ESM.pdf]

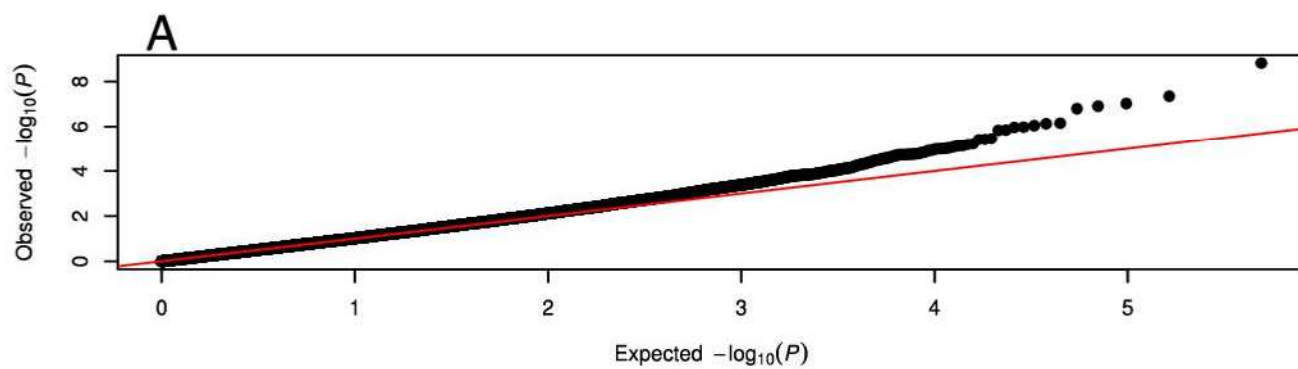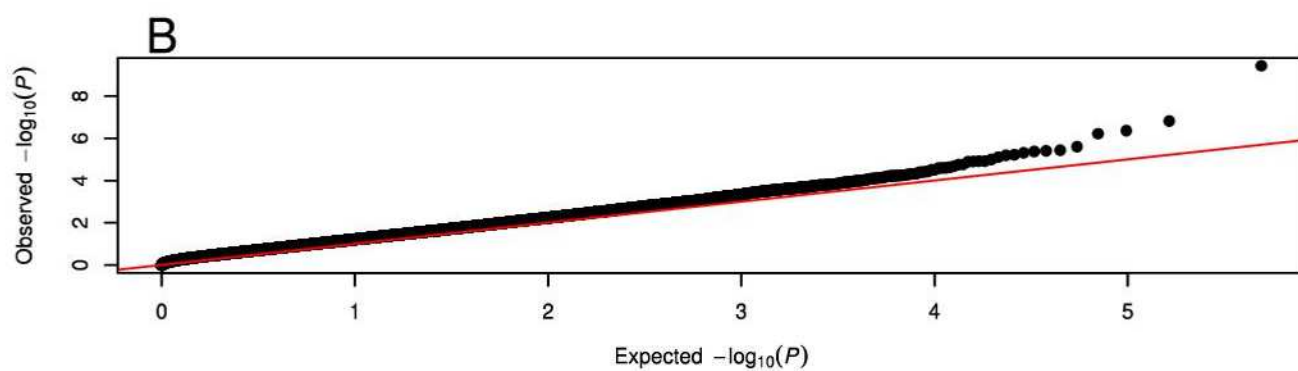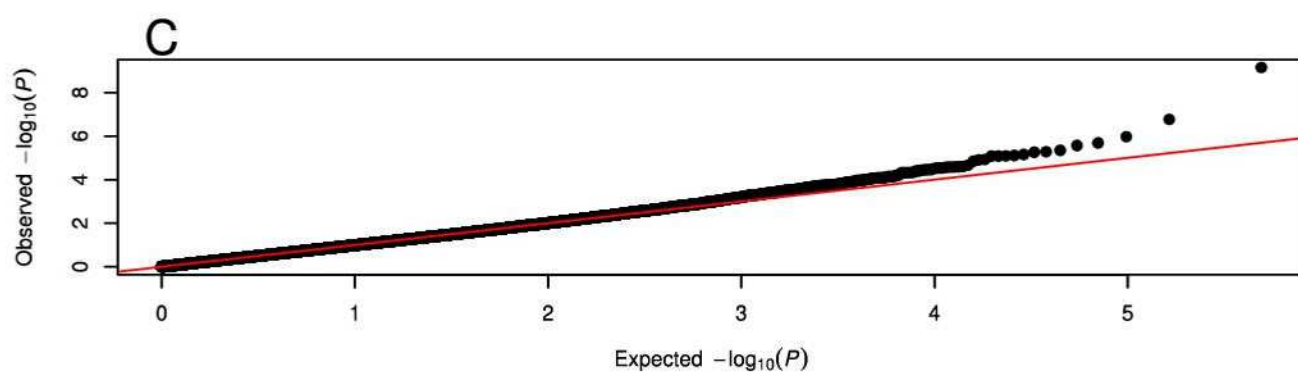

Supplement: Additional file 3 — Figure S3. Expected P values on the horizontal axis and observed P values on the vertical axis for the QQ plot analysis under the Fe-sufficient regime using 267 maize inbred lines of the association mapping population. The red line corresponds to a normal distribution. Traits with significant SNPs are represented: shoot water content (H 2O;A), root weight (RW;B), and SPAD value of leaf 3 (SP3;C). [file 12863_2014_153_MOESM3_ESM.pdf]

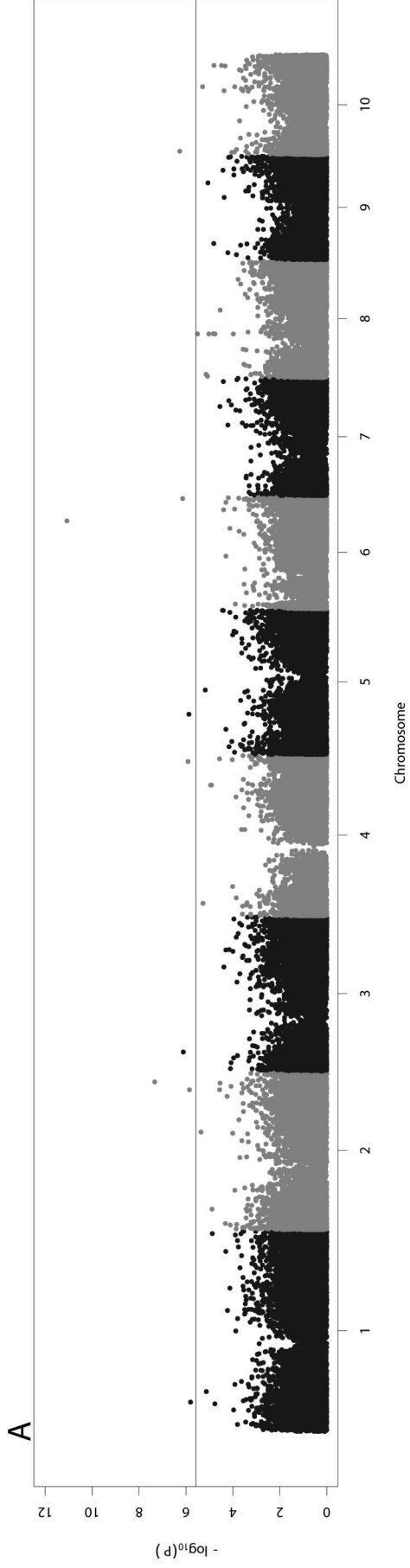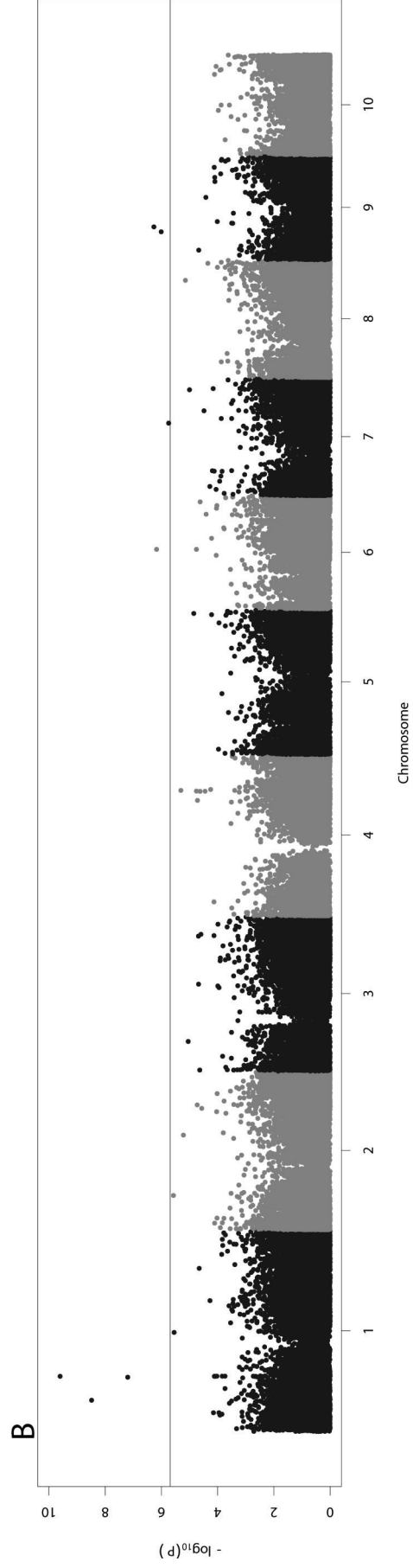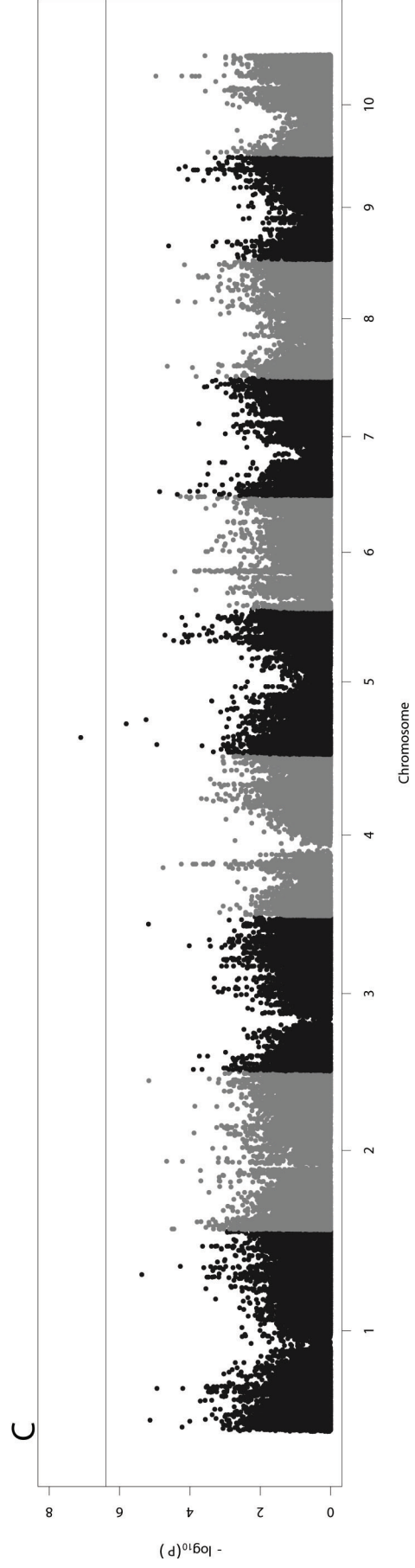

Supplement: Additional file 4 — Figure S2. Expected P values on the horizontal axis and observed P values on the vertical axis for the QQ plot analysis under the Fe-deficient regime using 267 maize inbred lines of the association mapping population. The red line corresponds to a normal distribution. Traits with significant SNPs are represented: leaf necrosis (NEC;A), root weight (RW;B), and shoot dry weight (SDW;C). [file 12863_2014_153_MOESM4_ESM.pdf]

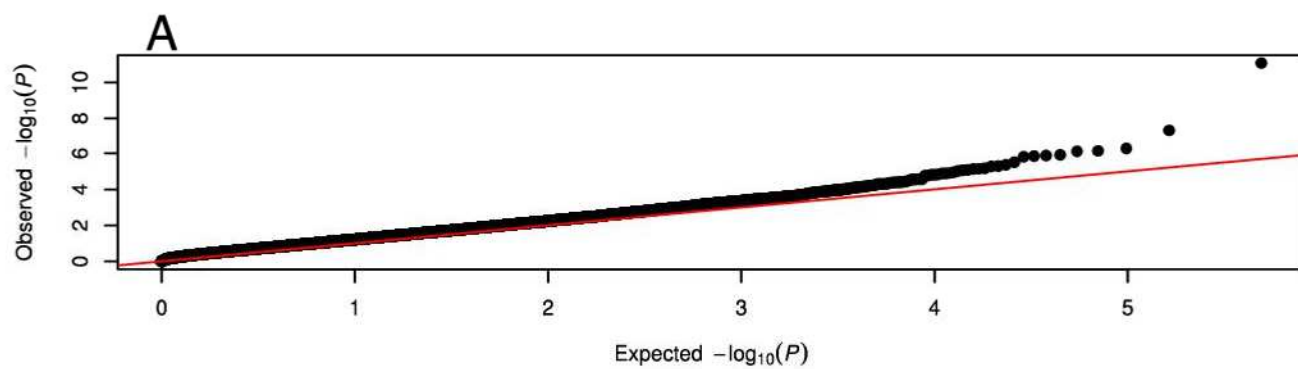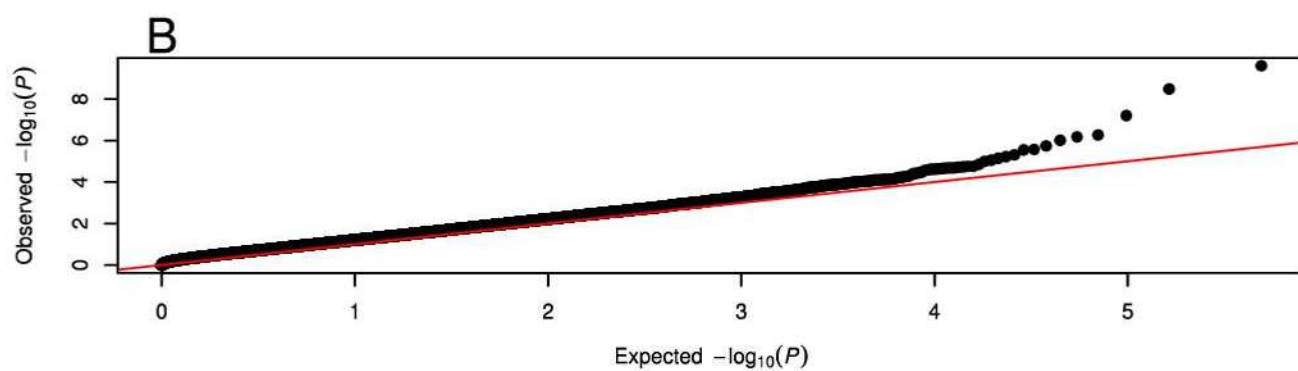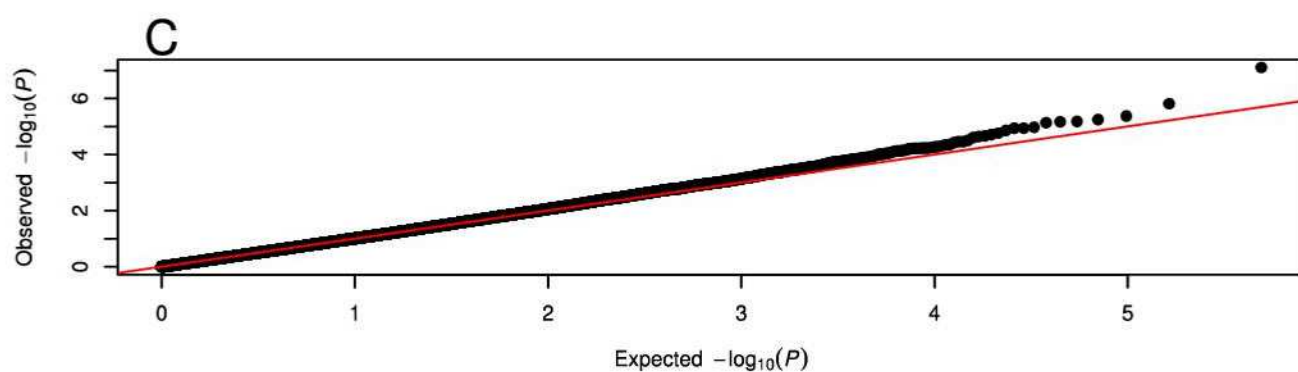

Supplement: Additional file 5 — Figure S4. Genes sequenced in our study that are reported in the literature to be involved in Fe-homeostasis of maize. [file 12863_2014_153_MOESM5_ESM.pdf]
